# Corpora amylacea deposition in the hippocampus of patients with mesial temporal lobe epilepsy: A new role for an old gene?

**DOI:** 10.4103/0971-6866.80358

**Published:** 2011-05

**Authors:** Abhijit Das, Shabeesh Balan, Anila Mathew, Venkataraman Radhakrishnan, Moinak Banerjee, Kurupath Radhakrishnan

**Affiliations:** 1 R. Madhavan Nayar Center for Comprehensive Epilepsy Care, Sree Chitra Tirunal Institute for Medical Sciences and Technology, Trivandrum, Kerala, India; 2 Human Molecular Genetics Laboratory, Rajiv Gandhi Center for Biotechnology, Trivandrum, Kerala, India

**Keywords:** *ABCB1*, corpora amylacea, epilepsy, genetics, hippocampal sclerosis, mesial temporal lobe epilepsy, P-glycoprotein, refractory epilepsy

## Abstract

**BACKGROUND:**

Mesial temporal lobe epilepsy (MTLE) is the most common medically refractory epilepsy syndrome in adults, and hippocampal sclerosis (HS) is the most frequently encountered lesion in patients with MTLE. Premature accumulation of corpora amylacea (CoA), which plays an important role in the sequestration of toxic cellular metabolites, is found in the hippocampus of 50–60% of the patients who undergo surgery for medically refractory MTLE-HS. However, the etiopathogenesis and clinical importance of this phenomenon are still uncertain. The *ABCB1* gene product P-glycoprotein (P-gp) plays a prominent role as an antiapoptotic factor in addition to its efflux transporter function. *ABCB1* polymorphism has been found to be associated with downregulation of P-gp expression. We hypothesized that a similar polymorphism will be found in patients with CoA deposition, as the polymorphism predisposes the hippocampal neuronal and glial cells to seizure-induced excitotoxic damage and CoA formation ensues as a buffer response.

**MATERIALS AND METHODS:**

We compared five single nucleotide polymorphisms in the *ABCB1* gene Ex06+139C/T (rs1202168), Ex 12 C1236T (rs1128503), Ex 17-76T/A (rs1922242), Ex 21 G2677T/A (rs2032582), Ex26 C3435T (rs1045642) among 46 MTLE-HS patients of south Indian ancestry with and without CoA accumulation.

**RESULTS:**

We found that subjects carrying the Ex-76T/A polymorphism (TA genotype) had a five-times higher risk of developing CoA accumulation than subjects without this genotype (Odds ratio 5.0, 95% confidence intervals 1.34-18.55; *P* = 0.016).

**CONCLUSION:**

We speculate that rs1922242 polymorphism results in the downregulation of P-gp function, which predisposes the hippocampal cells to seizure-induced apoptosis, and CoA gets accumulated as a buffer response.

Epilepsy is the most prevalent chronic neurological disorder and is a major public health concern, directly affecting an estimated 50 million people worldwide and involving an additional 500 million people as family members and caregivers of patients.[1] Despite the recent introduction of a range of new anti-epileptic drugs (AEDs) over the last 10 years, the problem of drug-resistant or drug-refractory epilepsy has not changed significantly, and 30% of the patients continue to be resistant to multiple AEDs.[2] Mesial temporal lobe epilepsy (MTLE) is the most common medically refractory epilepsy syndrome in adults, and hippocampal sclerosis (HS) is the most frequently encountered lesion in patients with MTLE.[3] The abundant premature accumulation of corpora amylacea (CoA) in the hippocampus has emerged as a distinctive marker of HS, which is seen in 50–60% of the subjects.[4,5] These are spherical, basophilic structures, which normally accumulate in the perivascular and subpial regions of the central nervous system as a result of aging and other neurodegenerative conditions like Alzheimer’s disease and Parkinson’s disease.[6] The extent of accumulation of CoA has been correlated with seizure duration and interictal psychosis in patients with MTLE-HS.[5] In this regard, the detection of CoA, a pathological marker of neurodegeneration, in the surgical specimens of patients with MTLE-HS is of special interest and deserves a closer scrutiny to elucidate the possible pathomechanisms of HS. Interestingly, the promotion of CoA formation has been linked to cellular stress, in particular oxidative stress and mitochondrial dysfunction.[6] As recurrent seizures induce significant oxidative stress in the hippocampus, it is likely that CoA formation ensues as a buffer response to seizure-induced excitotoxic damage in the hippocampal neuronal and glial cells.[5] P-glycoprotein (P-gp), an ATP-dependent efflux pump with a broad substrate specificity, encoded by the *ABCB1* gene, is postulated to mediate at least part of the drug resistance.[2,7] The expression of *ABCB1* in the epileptic foci of drug-resistant epilepsy was found to be several-fold elevated in both animal and human studies, indicating its significant role in epilepsy. The C3435T, a major allelic variant of the *ABCB1* gene, is proposed to play a crucial role in drug resistance in epilepsy. The C/C genotype carriers reportedly are at a higher risk of pharmacoresistance to AEDs, and this issue has been extensively investigated with conflicting results.[2] The recent meta-analyses have failed to confirm an association between the *ABCB1* C3435T polymorphism and the risk of drug resistance, suggesting a revision in contribution of this polymorphism in the multi-drug transporters hypothesis of drug resistance in epilepsy.[8–10] In addition to the efflux transporter function, P-gp plays an important role in tissue defence through the excretion of toxic compounds and their metabolites, especially in the presence of oxidative stress.[11,12] Hence, the overexpression of *ABCB1* gene products might be a tissue response to the oxidative stress of epilepsy. Uwai *et al*. reported an intron 16 polymorphism (Ex-76 T/A) that resulted in downregulation of P-gp function in renal carcinoma cells.[13] The Ex-76 T/A has been shown to be in linkage disequilibrium with G2677T and the alleles of Ex-76 T/A formed major haplotypes with G2677T and C3435T.[14] We hypothesized that a similar polymorphism will be found in patients with CoA deposition as the polymorphism predisposes the hippocampal neuronal and glial cells to seizure-induced excitotoxic damage and CoA formation ensues as a buffer response. This prompted us to investigate the role of *ABCB1* polymorphisms in the premature accumulation of CoA by comparing the distribution of single nucleotide polymorphisms (SNPs) between groups of MTLE-HS patients with and without CoA in their hippocampus.

## Materials and Methods

### 

#### Selection of subjects

The epilepsy patients were recruited through the Epilepsy Clinic at R. Madhavan Nayar Center for Comprehensive Epilepsy Care, Sree Chitra Tirunal Institute of Medical Sciences and Technology at Trivandrum, India. Phenotypic definition of drug resistance is a critical issue in epilepsy genetics. There is no consistent clinical definition of multidrug resistance in epilepsy, and agreement among the different definitions is strong but imperfect.[15] This has resulted in diverse criteria used by different researchers, or even a lack of explicit criteria in some cases, rendering it difficult to compare findings across studies. To address this issue and to avoid population substructuring in the patient population, we used stringent patient selection criteria to maintain genotype homogeneity. Only patients with self-declared Malayalam-speaking south Indian ancestry with pathologically proven HS were recruited who were seizure-free for more than 1 year after surgery. We recruited 46 consecutive patients with MTLE who had undergone anterior temporal lobectomy and had pathologically verified HS. The details of our noninvasive presurgical evaluation protocol have been described previously.[16] We excluded subjects with HS associated with temporal lobe neoplasms (secondary HS).

#### Surgical procedure

For MTLE-HS, we performed an *en bloc* standard anterior temporal lobectomy with amygdalohippocampectomy.[16] After a standard temporal craniotomy, under general anesthesia, which exposed approximately 8 cm of the temporal lobe and the inferior frontal gyrus, a 5–6-cm block of temporal lateral cortex along with superior temporal gyrus was resected depending on temporal lobe dominance and the presence of prominent cortical vessels. The anterior two-thirds of the hippocampus and the lateral two-thirds of the amygdala were resected *en bloc* along with the uncus and parahippocampal gyrus by subpial dissection using the operating microscope.

#### Pathological examination

**Figure 1:**
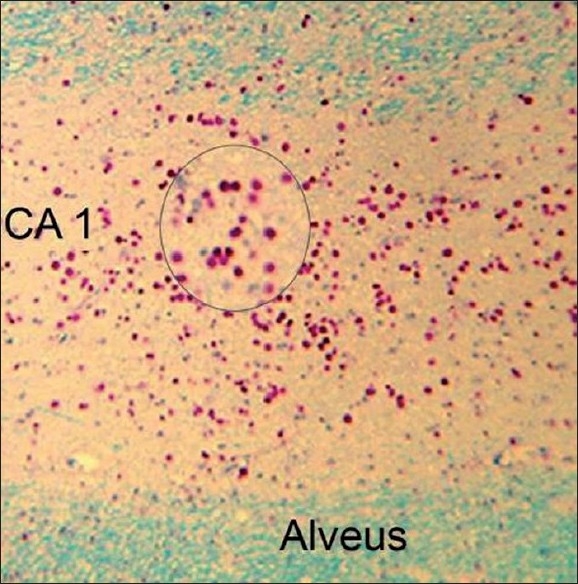
Microphotograph showing dense deposition of corpora amylacea (CoA) in the CA1 sector of the hippocampus in a 42-year-old male with mesial temporal lobe epilepsy and hippocampal sclerosis. Inset shows a magnified view of the CoA (Luxol-fast blue–Periodic Acid Schiff stain, X150). 83 mm × 84 mm (300 × 300 DPI)

Four-micrometer-thick histological sections of the resected temporal lobe were generated from 10% formalin-fixed, paraffin-embedded tissue. In addition to the routine hematoxylin and eosin stain, we stained the hippocampal sections with Luxol fast blue–Periodic acid Schiff’s stain to highlight the presence of CoA in the hippocampus and in the adjoining temporal structures [Figure 1]. We defined HS as the loss of neuronal cell population of ≥30% in the CA1 sector of the hippocampal formation with or without neuronal loss and gliosis involving other mesial temporal lobe structures.[4] Based on the density of CoA in the CA1 sector of the hippocampus, all the specimens were scored on a semiquantitative scale as: >10 CoA per high-power field (HPF) as grade 3, 6–10/HPF as grade 2 and ≤5/HPF as grade 1.[4] Subjects with grade 1, grade 2 and grade 3 CoA deposition in the hippocampi formed the MTLE-HS-CoA +ve group and subjects without demonstrable CoA anywhere in the specimen formed the MTLE-HS-CoA –ve group. For this study, we did not take into consideration the extent of the neuronal loss or the pattern of distribution of CoA in the hippocampus or extrahippocamal structures.

#### Genotyping

We augmented the reported polymorphism with the addition of four SNPs flanking the C3435T, which together capture the majority of the functional variations across *ABCB1* Ex06+139C/T (rs1202168), Ex 12 C1236T (rs1128503), Ex 17-76T/A (rs1922242), Ex 21 G2677T/A (rs2032582) and Ex26 C3435T (rs1045642) [Table 1]. The genotyping was carried out at the Department of Human Molecular Genetics, Rajiv Gandhi Center for Biotechnology, Trivandrum, India. Blood samples were obtained after informed consent and the study was approved by the Institutional Ethics Committee for biomedical subjects as per the Indian Council of Medical Research (ICMR) guidelines.[17] Blood samples (5 ml) were drawn into EDTA vials and genomic DNA was isolated from human leukocytes by standard methods. Polymerase chain reaction (PCR) was carried out in a total volume of 10 μl containing 50 ng DNA, 250 μM dNTP (Amersham Pharmacia Biotech, Uppsala, Sweden), 10 pmol of each primer (Table), 1X PCR buffer (Bangalore Genei, Bangalore, India) and 0.5 U Taq polymerase enzyme (Bangalore Genei). PCR was programmed for initial denaturation at 95°C for 5 min, followed by 35 cycles of denaturation at 95°C for 30s, annealing for 30 s (depending on the primer) and extension at 72°C for 30 s in a MJ Research PTC 200 Thermal Cycler [Table 1]. PCR products were digested with restriction enzymes (depending on the nature of polymorphism) according to the manufacturer’s protocol (New England Biolab, Ipswich, MA, USA), separated on a 3% agarose gel and stained with ethidium bromide for visualization.

**Table 1:** Details of methodology used in genotyping

SNP ID	Location	Primer sequence 5’–3’	Tm (ºC)	Enzyme	RFLP pattern
Ex06+139C/T rs1202168	chr7:87033898	AGGTTTCATTTTGGTGCCTG	61.5	SspI	299* 275, 24#
		GAACAAAAGGATGCACACGACA			
Ex 12 C1236T rs1128503	chr7:87017537	TACCTGTGTCTGTGAATTGCC	59.3	HaeIII	269, 62, 35*
		CCTGACTCACCACACCAATG			269, 97#
Ex 17-76T/A rs1922242	chr7:87011603	TTTGTCAACATTTTTTTGAAGC	69.2	ApoI	231, 84* 120, 111, 84#
		TATTATTGCAAATGCTGGTTGC			
Ex 21G2677T/A rs2032582	chr7:86998554	TGCAGGCTATAGGTTCCAGG	67.9	BanI	198, 26*
		TTTAGTTTGACTCACCTTCCC			224#
Ex26 C3435T rs1045642	chr7:86976581	TGTTTCAGCTGCTTGATGG	59.4	Sau3AI	158, 39*
		AAGGCATGTATGTTGGCCTC			197#

* wild type

# mutant

#### Statistical methods

We summarized the quantitative data as mean ± standard deviation (SD). Chi square-tests, Fisher’s exact tests and t-tests were used to evaluate the statistical significance of the difference in clinical characteristics between the MTLE-HS-CoA+ and MTLE-HS-CoA– patient groups. A *P*-value of ≤0.05 was considered as significant. The frequency distribution of allele and genotype frequencies of the five polymorphisms was analyzed in the MTLE-HS-CoA+ and MTLE-HS-CoA– patients in a case-control format using the statistical software UNPHASED (version 2.404). The method for case-control data is a standard unconditional logistic regression identical to the model-free method T5 of EHPLUS and the log-linear modelling of Mander.[18] Binary logistic regression was used to adjust for age, sex and seizure duration. Association was expressed as odds ratios (OR) or risk estimates with 95% confidence intervals (CI). Association was considered significant at *P* <0.05. All analyses were performed using SPSS statistical analysis software, Version 17.0 (SPSS, Chicago, IL, USA).

## Results

### 

#### Patient characteristics

Of the 46 (26 male and 20 female) MTLE-HS patients who were included in the study, 24 (52.1%) were MTLE-HS-CoA+ and 22 (47.9%) were MTLE-HS-CoA–. The mean age at surgery was 32.3 years (range, 17–50 years) and the mean duration of epilepsy prior to surgery was 22.8 years (range, 6–47 years). Antecedent history of febrile seizures was present in 19 (41.3%) patients. In the MTLEHS-CoA+ group, there were 11 patients (45.9%) with grade 1, seven (29.1%) patients with grade 2 and six (25%) patients with grade 3 CoA accumulation. The distribution of variables among the MTLE-HS-CoA+ and the MTLE-HS-CoA– groups of patients is compared in Table 2, and is elaborated.

**Table 2:** Clinical characteristics of patients

Characteristics	MTLE-HS-CoA+ (n=24)	MTLE-HS-CoA- (n=22)	*P*-value
Male:female	13:11	13:9	NS
Age at surgery (year, mean ± SD)	36.42 ± 7.14	27.86 ± 7.64	<0.0005
Duration of epilepsy	25.73 ± 10.63	19.56 ± 10.44	0.054
Seizure frequency/year	123.16 ± 48.99	180.80 ± 92.50	NS
History of febrile seizures (%)	17 (70.8)	12 (54.5)	NS

MTLE-HS-CoA+ = Mesial temporal lobe epilepsy with hippocampal sclerosis with corpora amylacea; MTLE-HS-CoA- = Mesial temporal lobe epilepsy with hippocampal sclerosis without corpora amylacea; NS = Not significant; SD = Standard deviation

#### Clinical features

The age at surgery was found to be significantly higher for the MTLE-HS-CoA+ patients compared with the MTLE-HS-CoA– patients (36.42 ± 7.14 years versus 27.86 ± 7.64 years, *P* < 0.0005). The duration of epilepsy before surgery was also borderline significantly longer in the MTLE-HS-CoA+ patients compared with the MTLE-HS-CoA– patients (25.73 ± 10.63 years versus 19.56 ± 10.44 years, *P* = 0.054). However, none of the other variables such as age at onset of habitual seizures, seizure frequency, seizure types, history of antecedent events, family history of seizures, presence of mental retardation or other co-morbid conditions and AED use profile differed significantly between the groups.

#### Genotypic analysis

Patients in the MTLE-HS-CoA+ group were more likely to have the TA genotype at *ABCB1* Ex -76T/A compared with those in the MTLE-HS-CoA– group (70% versus 31.8%, *P* = 0.016, OR = 5, 95% CI = 1.34-18.5) [Table 3]. Significant differences were observed at the allele level also (*P* = 0.04). After adjustment of the odds ratios for age at surgery, sex, age at onset, duration of epilepsy and genotype frequency, there was a significant difference between the MTLE-HS-CoA+ and the MTLE-HS-CoA– groups (*P* = 0.022, OR = 6.4, 95% CI = 1.3-31.8) for the TA genotype. The genotype frequency of the 2677G>T/A *ABCB1* polymorphism also differed significantly between the MTLE-HS-CoA+ and the MTLE-HS-CoA– groups (*P* = 0.02, OR = 16.6, 95% CI = 1.36-204). Significant differences were observed at the allele level also (*P* = 0.01). However, after adjustment of the ORs for age at surgery, sex, age at onset, duration of epilepsy and genotype frequency, there was no significant difference between the MTLE-HS-CoA+ and the MTLE-HS-CoA– groups. Similarly, genotype frequencies of the 1236C>T, +139C>T and C3435T *ABCB1* polymorphism also did not differ significantly between the MTLE-HS-CoA+ and the MTLE-HS-CoA– groups. However, there was a significant difference at the allele level for the allele frequency of +139C>T (*P* = 0.03).

**Table 3:** Comparison of genotype and allele frequencies between patients with mesial temporal lobe epilepsy with (CoA+) and without (CoA-) corpora amylacea in the hippocampus

Group	Genotype frequency (%)			Allele frequency (%)		
	Ex-76 T/A					
	TT	TA	AA	T		A
CoA+	6 (30)	14 (70)	0	26 (65)		14 (35)
CoA	15 (68.18)	7 (31.82)	0	37 (84.09)		7 (15.91)
*P*-value		0.016*			0.04	
	G2677T					
	GG	GT	TT	G		T
CoA+	6 (25.00)	15 (62.50)	3 (12.50)	27 (56.25)		21 (43.75)
CoA	1 (4.55)	11 (50.00)	10 (45.45)	13 (29.55)		31 (70.45)
*P*-value		0.02			0.01	
	C3435T					
	CC	CT	TT	C		T
CoA+	0	11 (45.83)	13 (54.17)	11 (22.92)		37 (77.08)
CoA	0	7 (31.82)	15 (68.18)	7 (15.91)		37 (84.09)
*P*-value		0.33			0.4	
	C1236T					
	CC	CT	TT	C		T
CoA+	2 (8.33)	10 (41.67)	12 (50.00)	14 (29.17)		34 (70.83)
CoA	3 (13.64)	5 (22.73)	14 (63.64)	11 (25)		33 (75)
*P*-value		0.38			0.65	
	+139C/T					
	CC	CT	TT	C		T
CoA+	4 (16.67)	18 (75.00)	2 (8.33)	26 (54.17)		22 (45.83)
CoA	1 (4.55)	12 (54.55)	9 (40.91)	14 (31.82)		30 (68.18)
*P*-value		0.06			0.03	

* Significant in logistic regression analysis. Odd’s ratio (OR) = 5, 95% confidence intervals (CI) = 1.34–18.55

## Discussion

In this study, using a strict phenotypic definition, we found an association between the intronic polymorphic variation (TA genotype in Ex17-76) in the *ABCB1* gene with the CoA deposition in MTLE-HS in a cohort of south Indian epilepsy patients. This is a novel association for the *ABCB1* gene, but its role in CoA deposition can have a biologic plausibility. *ABCB1* belongs to the broad group of transporters known as ATP-binding cassette (ABC) transporter of the MDR/TAP subfamily.[19] The localization of ABC transporters in organs, with a barrier function, and the broad substrate specificities suggest an important role in tissue defense. The defense mechanism formed by ABC transporters under physiological circumstances is directed against the accumulation of potentially harmful compounds.

Interestingly, in a situation of organ damage or disease, changes in the expression levels of ABC transporters have been observed, probably to compensate for the increased load of harmful products of oxidative stress formed during an insult or to compensate for the loss of efflux pumps in damaged tissue.[12] In addition to its protective function, P-gp has also been implicated in resistance to apoptosis, most importantly by inhibiting Fas-induced caspase-3 activation and by modulating ceramide metabolism.[12,20] Hence, the overexpression of *ABCB1* gene products at the site of tissue damage is a physiological phenomenon for tissue defense. Overexpression of *ABCB1* in the epileptic tissue, both in animal and human tissue, was one of the key evidence to propose its role in drug resistance in epilepsy.[21,22] The expression of *ABCB1* in the epileptic foci of drug-resistant epilepsy patients was shown to be elevated 10-fold.[23] Elevated levels of *ABCB1* and multidrug-associated protein (ABCC1/MRP1) have also been associated with pathologies associated with drug refractoriness, such as in tuberous sclerosis and also in epileptic tissue surrounding dysembryoplastic neuroepithelial tumors, focal cortical dysplasia and hippocampal sclerosis. The adjacent nonepileptic tissue from the same patients did not show elevated expression of the transporter.[24] In 2003, Siddiqui *et al*.[25] reported the C3435T polymorphism in the *ABCB1* gene as being associated with resistance to multiple AEDs, and leading to the suggestion, for the first time, that drug resistance in epilepsy might be genetically determined. Since then, several other genetic association studies have attempted to verify this result; however, taken overall, the role of P-gp in drug resistance in epilepsy still remains uncertain.[2] Three recent meta-analyses addressed the issue.[8–10] The first report in 1073 Caucasians patients included three studies with the same definition of drug responsiveness and drug resistance,[8] and the second one included 11 studies involving 3371 patients with different ethnicities, but the same definition of drug responsiveness and drug resistance.[9] Neither of these studies could confirm this association and stratification of ethnic subgroups in the second meta-analysis also provided no further evidence. The findings of the third meta-analysis indicate that neither the C allele nor the T allele carriers of the *ABCB1* C3435T polymorphism confer significant risk to drug resistance in epilepsy. In the subgroup analysis for the Asian and the Caucasian populations, none of the genetic comparisons showed a significant association. Hence, the substitution of C to T at position 3435 of the exon 26 of the *ABCB1* gene does not effect the response to AEDs in the epilepsy patients with different ethnicities. Even in subgroup meta-analyses based on the new definition of drug resistance by International League Against Epilepsy (ILAE)[15] and ethnicity, to address the issue of different definitions of drug responsiveness and drug resistance in the patients with various ethnicities, did not show any association under all genetic models.[10] Hence, in view of these data, we may have to revise the role for *ABCB1* gene in epilepsy and more importance must be attributed to its role in tissue defense in epileptic tissue. The role of CoA in sequestration of toxic cellular metabolites is being increasingly recognized and is supported by the presence of ubiquitin and heat shock proteins in CoA and its abundant accumulation in neurodegenerative diseases, where oxidative stress and mitochondrial dysfunction are common denominators compared with age-matched controls.[6] In patients with MTLE-HS, premature accumulation of CoA in the hippocampus might be a reflection of a tissue reaction to buffer the free radicals and other toxic metabolites generated as a result of repeated seizures. Our observation in this and previous studies[5] of the association between older age and longer duration of epilepsy with CoA accumulation in the hippocampus is in agreement with this hypothesis. Is there a relationship between *ABCB1* polymorphisms and CoA accumulation that we observed in our well-characterized MTLE-HS patients? We hypothesize a link through the tissue defense function of *ABCB1*-encoded P-gp. The Ex-76 T/A has been shown to be in linkage disequilibrium with G2677T and the alleles of Ex-76 T/A formed major haplotypes with G2677T and C3435T.[14] Although data to date are mainly available for coding SNPs in exons 21 and 26, Uwai *et al*.[13] found that Ex-76 T/A (intron 16) polymorphism resulted in downregulation of P-gp function in renal carcinoma cells. We hypothesize that downregulation of P-gp function predisposes the hippocampal neuronal and glial cells to seizure-induced excitotoxic damage and CoA formation ensues as a buffer response. Our results demonstrate that *ABCB1* polymorphisms are associated with CoA deposition in MTLE-HS patients in south Indian patients, and further substantiate the need to revise its role in epilepsy genetics. However, the association found in our study is modest, most likely due to the small sample size. As the stringent selection criteria resulted in the small sample size, haplotype analysis was not possible. As the G2677T polymorphism (genotypic and allelic) was also found to be associated with CoA deposition and the Ex-76 T/A has been shown to be in linkage disequilibrium with G2677T, the haplotypic analysis would be interesting. Hence, a further study with a larger sample size and P-glycoprotein functional expression studies will be required to establish the relationship between Ex-76 T/A polymorphism and CoA accumulation.

## References

[1] Radhakrishnan K (2009). Challenges in the management of epilepsy in resource-poor countries. Nat Rev Neurol.

[2] Löscher W, Klotz U, Zimprich F, Schmidt D (2009). The clinical impact of pharmacogenetics on the treatment of epilepsy. Epilepsia.

[3] (2004). ILAE Commission Report. Mesial temporal lobe epilepsy with hippocampal sclerosis. Epilepsia.

[4] Radhakrishnan VV, Rao MB, Radhakrishnan K, Thomas SV, Nayak DS, Santoshkumar B (1999). Pathology of temporal lobe epilepsy: An analysis of 100 consecutive surgical specimens from patients with medically refractory epilepsy. Neurol India.

[5] Radhakrishnan A, Radhakrishnan K, Radhakrishnan VV, Mary PR, Kesavadas C, Alexander A (2007). Corpora amylacea in mesial temporal lobe epilepsy: Clinicopathological correlations. Epilepsy Res.

[6] Cavanagh JB (1999). Corpora-amylacea and the family of polyglucosan diseases. Brain Res Rev.

[7] Löscher W, Potschka H (2005). Drug resistance in brain diseases and the role of drug efflux transporters. Nat Rev Neurosci.

[8] Leschziner GD, Andrew T, Leach JP, Chadwick D, Coffey AJ, Balding DJ (2007). Common *ABCB1* polymorphisms are not associated with multidrug resistance in epilepsy using a gene-wide tagging approach. Pharmacogenet Genomics.

[9] Bournissen FG, Moretti ME, Juurlink DN, Koren G, Walker M, Finkelstein Y (2009). Polymorphism of the MDR1/*ABCB1* C3435T drug-transporter and resistance to anticonvulsant drugs: A meta-analysis. Epilepsia.

[10] Haerian BS, Roslan H, Raymond AA, Tan CT, Lim KS, Zulkifli SZ (2010). *ABCB1* C3435T polymorphism and the risk of resistance to antiepileptic drugs in epilepsy: A systematic review and meta-analysis. Seizure.

[11] Johnstone RW, Ruefli AA, Smyth MJ (2000). Multiple physiological functions for multidrug transporter p-glycoprotein?. Trends Biochem Sci.

[12] Huls M, Russel FG, Masereeuw R (2009). The role of ATP binding cassette transporters in tissue defense and organ regeneration. J Pharmacol Exp Ther.

[13] Uwai Y, Masuda S, Goto M, Motohashi H, Saito H, Okuda M (2004). Common single mucleotide poymorphisms of the MDR1 gene have no influence on its mRNA expression level of normal kidney cortex and renal cell carcinoma in Japanese nephrectomized patients. J Hum Genet.

[14] Potočnik U, Ferkolj I, Glavač D, Dean M (2004). Polymorphisms in multidrug resistance 1 (MDR1) gene are associated with refractory Crohn disese and ulcerative colitis. Genes and Immunity.

[15] Kwan P, Arzimanoglou A, Berg AT, Brodie MJ, Allen Hauser W, Mathern G (2010). Definition of drug resistant epilepsy: Consensus proposal by the ad hoc Task Force of the ILAE Commission on Therapeutic Strategies. Epilepsia.

[16] Rao MB, Radhakrishnan K (2000). Is epilepsy surgery possible in countries with limited resources?. Epilepsia.

[17] (2006). Indian Council of Medical Research. Ethical Guidelines for Biomedical Research on Human Subjects. http://icmr.nic.in/ethical_guidelines.pdf.

[18] Dudbridge F (2003). Pedigree disequilibrium tests for multilocus haplotypes. Genet Epidemiol.

[19] Aller SG, Yu J, Ward A, Weng Y, Chittaboina S, Zhuo R (2009). Structure of P-glycoprotein reveals a molecular basis for poly-specific drug binding. Science.

[20] Pallis M, Turzanski J, Higashi Y, Russell N (2002). P-glycoprotein in acute myeloid leukaemia: Therapeutic implications of its association with both a multidrug-resistant and an apoptosis-resistant phenotype. Leuk Lymphoma.

[21] Rizzi M, Caccia S, Guiso G, Richichi C, Gorter JA, Aronica E (2002). Limbic seizures induce P-glycoprotein in rodent brain: Functional implications for pharmacoresistance. J Neurosci.

[22] van Vliet EA, van Schaik R, Edelbroek PM, Voskuyl RA, Redeker S, Aronica E (2007). Region-specific overexpression of P-glycoprotein at the blood-brain barrier affects brain uptake of phenytoin in epileptic rats. J Pharmacol Exp Ther.

[23] Marchi N, Guiso G, Rizzi M, Pirker S, Novak K, Czech T (2005). A pilot study on brain to plasma partition of 10, 11-dyhydro-10-hydroxy-5H-dibenzo(b,f)azepine-5-carboxamide and MDR1 brain expression in epilepsy patients not responding to oxcarbazepine. Epilepsia.

[24] Tishler DM, Weinberg KI, Hinton DR, Barbaro N, Annett GM, Raffel C (1995). MDR1 gene expression in brain of patients with medically intractable epilepsy. Epilepsia.

[25] Siddiqui A, Kerb R, Weale ME, Brinkmann U, Smith A, Goldstein DB (2003). Association of multidrug resistance in epilepsy with a polymorphism in the drug-transporter gene *ABCB1*. N Engl J Med.

